# Dynamic regulation of uncoupling protein 2 content in INS-1E insulinoma cells

**DOI:** 10.1016/j.bbabio.2008.07.001

**Published:** 2008-10

**Authors:** Vian Azzu, Charles Affourtit, Eamon P. Breen, Nadeene Parker, Martin D. Brand

**Affiliations:** MRC Dunn Human Nutrition Unit, Wellcome Trust/MRC Building, Hills Road, Cambridge, CB2 0XY, UK

**Keywords:** ANT, adenine nucleotide translocase, GSIS, glucose-stimulated insulin secretion, FCS, fetal calf serum, Q, glutamine, ROS, reactive oxygen species, UCP, uncoupling protein, rUCP2, recombinant UCP2, Glutamine, Half-life, Mitochondria, Pancreatic beta-cell, Serum starvation, UCP2

## Abstract

Uncoupling protein 2 (UCP2) regulates glucose-stimulated insulin secretion in pancreatic beta-cells. UCP2 content, measured by calibrated immunoblot in INS-1E insulinoma cells (a pancreatic beta-cell model) grown in RPMI medium, and INS-1E mitochondria, was 2.0 ng/million cells (7.9 ng/mg mitochondrial protein). UCP2 content was lower in cells incubated without glutamine and higher in cells incubated with 20 mM glucose, and varied from 1.0–4.4 ng/million cells (2.7–14.5 ng/mg mitochondrial protein). This dynamic response to nutrients was achieved by varied expression rates against a background of a very short UCP2 protein half-life of about 1 h.

## 1 Introduction

Pancreatic beta-cells maintain glycaemic control by secreting insulin in response to high glucose concentrations. Mitochondria play an important role in glucose-stimulated insulin secretion (GSIS) by coupling glucose oxidation to ATP production. Glucose oxidation raises the mitochondrial protonmotive force and increases the ATP:ADP ratio, leading to closure of ATP-sensitive potassium channels, plasma membrane depolarisation, activation of voltage-gated calcium channels, and an influx of calcium that promotes exocytosis of insulin-containing granules [1]. This acute phase of insulin secretion is followed by a second sustained phase driven by mitochondrial and cytosolic amplifying signals [2,3].

Uncoupling protein 2 (UCP2) attenuates insulin secretion by regulated uncoupling, which lowers the mitochondrial protonmotive force and the cytosolic ATP:ADP ratio. *Ucp2* knockout in mice [4] or its acute removal by siRNA knockdown in whole animals [5] and beta-cell lines [6] improves GSIS, and overexpression of UCP2 in isolated islets [7] or whole animals [8] attenuates GSIS. UCP2 activity may coordinate the physiological response of beta-cells to fluctuating nutrient supply [9].

UCP2 content in beta-cells is regulated at the transcriptional and translational levels. *Ucp2* gene expression is upregulated by hyperglycaemia and hyperlipidaemia, via transcription factors including sterol regulatory element binding protein 1c (SREBP 1c) [10], and peroxisome proliferator-activated receptors PPAR-alpha [11] and PPAR-gamma [12,13]. Cold exposure, acting via sympathetic nerve stimulation and the PPAR-gamma coactivator PGC-1, can also increase *Ucp2* gene expression [14]. Repression of transcription, on the other hand, has been shown to involve sirtuins [15,16] and interleukin IL-1-beta [17]. In other cell types, *Ucp2* mRNA levels do not necessarily correlate with protein concentrations [18], because of translational regulation. An upstream open reading frame inhibits translation of *Ucp2* mRNA, and inhibition can be overcome by glutamine [19,20]. UCP2 can also be regulated at the functional level; it is activated by reactive oxygen species in beta-cell mitochondria [21] and beta-cells [22], and inhibited by purine nucleoside di- and tri-phosphates [21]. Nothing is known about UCP2 protein degradation in beta-cells, although in ovarian and granulosa cell lines, the half-life of UCP2 is less than 1 h [23].

In the present paper we characterise the content and regulation of UCP2 in INS-1E insulinoma cells, a widely used clonal rat pancreatic beta-cell model. We show that UCP2 concentration varies 4–5-fold depending on the composition of the growth medium, because of translational and possibly transcriptional control. The half-life of UCP2 is about 1 h, allowing rapid and dynamic fluctuations in the UCP2 content of the cells in response to nutrients in the medium.

## 2 Materials and methods

### 2.1 Sample preparation

All chemicals were from Sigma-Aldrich or Gibco unless stated otherwise. INS-1E cells were maintained in RPMI medium as described in [24]. For quantification of UCP2 protein and mRNA, Nunclon™ 500 cm^2^ trays were seeded at 2.5 × 10^5^ cells/ml and grown to approximately 90% confluence. Following two washes with ice-cold SHE buffer (0.25 M sucrose, 20 mM HEPES (pH 7.4), 2 mM EGTA, 10 mM KCl, 1.5 mM MgCl_2_ and 0.1% (w/v) defatted bovine serum albumin), cells were scraped off the trays and harvested by centrifugation at 350 *g* (10 min, 4 °C), then aliquots were made for cell UCP2 or mRNA quantification or mitochondrial isolation. Mitochondria were prepared from INS-1E cells as described in [25], either with or without a protease inhibitor cocktail.

Mitochondria from rat and mouse tissues were isolated by standard methods. Female Wistar rats (Charles River Laboratories, UK) and *Ucp2*^−/−^ [4] and wild-type sibling paired mice were 10–12 months old. *Ucp2* knockout was confirmed by PCR analysis of the *Ucp2* locus and immunoblotting. Animals were housed at 21 ± 2 °C, humidity 57 ± 5% with a 12 h light/dark cycle. Food and water were available *ad libitum*. Home Office Guidelines for the Care and Use of Laboratory Animals (UK) were followed.

### 2.2 Quantification of UCP2 protein

Recombinant UCP2 (rUCP2) protein standards were prepared by subcloning, overexpression and purification of rat UCP2. cDNA containing the rat *Ucp2* gene was from imaGenes (Berlin, Germany). The complete UCP2 transcript was obtained by PCR using UCP2 forward (5′-CACCATGGTTGGTTTCAAGGCCACCGATGT-3′) and UCP2 reverse (5′-TCAAAAGGGTGCCTCCCGGGATTCATAGG-3′) primers. The transcript beginning CACC was subcloned into pET100/D-TOPO (Invitrogen, Paisley, UK) using the TOPO^®^ cloning system according to the manufacturer's instructions. This plasmid added an N-terminal appendage (including a His_6_-tag) to the expressed protein. BL21 Star transformed with pET100/D-TOPO containing *Ucp2* were grown at 37 °C until OD_600_ = 0.5. Expression was induced using 1 mM IPTG for 6 h. Inclusion bodies containing rUCP2 were isolated as described [26] and solubilised in 50 mM HEPES pH 7.4 containing 8 M urea and 25 mM DTT for 1 h at room temperature. The sample was diluted 10-fold then purified using a HisTrap HP™ column (GE Healthcare, UK) according to the manufacturer's instructions. The concentration of eluted protein was determined by BCA protein assay (Pierce) and purity was assessed by SDS PAGE. SDS PAGE, immunoblotting and quantification of UCP2 in cells and mitochondria were performed as described in [6]. Where appropriate, membranes were stripped (Restore™ Plus, Pierce) and reblotted for adenine nucleotide translocase (ANT), or stained with Gelcode^®^ Blue reagent (Pierce) to quantify protein. UCP2 (sc-6525) and ANT (sc-9300) antibodies were from Santa Cruz Biotechnology, USA.

### 2.3 Quantification of *Ucp2* transcript

Total RNA was extracted from snap-frozen pellets of (0.5–1) × 10^5^ INS-1E cells using RNeasy kit (Qiagen) and cDNA was synthesised using random hexamers and the Transcriptor First Strand cDNA Synthesis Kit (Roche) according to the manufacturer's protocol. *Ucp2* template was amplified (UCP2 forward: 5′-GATCTCATCACTTTCCCTCTAGACA-3′, UCP2 reverse: 5′-CCCTTGACTCTCTCCTTGG-3′, UCP2 probe: 5′-6CGCCAAAGTCCGGCTGCAGA0-3′) using a 7900HT Fast Real-Time PCR System with Taqman Master Mix (ABI). Expression was normalised to 18S rRNA (18S forward: 5′-CGGCTACCACATCCAAGGAA-3′, 18S reverse: 5′-GCTGGAATTACTGTGGCT-3′, 18S probe: 5′-6GAGGGCAAGTCTGGTGCCAG0-3′) under each condition.

### 2.4 UCP2 half-life

INS-1E were seeded at 3 × 10^5^ cells/ml in 24-well plates (Falcon) and used after 24 h in fully supplemented RPMI medium containing 2 mM, 10 mM or 20 mM glucose. Cells were treated with 10 μg/ml cycloheximide at time zero to arrest protein translation. At various time points they were harvested by trypsinisation in RPMI containing protease inhibitors and pelleted by centrifugation (800 *g*, 2 min). Cells (or isolated mitochondria) were resuspended in gel loading buffer, boiled for 5 min and vortexed vigorously. UCP2 content was determined by immunoblot.

### 2.5 Data analysis

Data are presented as means ± SEM or range. Differences were tested for statistical significance by one-way ANOVA applying Dunnett's post hoc analysis. Values of *P *< 0.05 were considered significant.

## 3 Results and discussion

### 3.1 Dynamic variation of UCP2 content

Characterisation of INS-1E cells has shown that they retain GSIS and a high degree of differentiation, making them a good model of pancreatic beta-cells [27]. Initial observations suggested that UCP2 content was high in INS-1E cells grown in standard RPMI medium, but much lower in cells incubated in simple salts medium for 90 min (C. Affourtit & M.D. Brand, unpublished observations). To investigate why, we used immunoblotting calibrated with recombinant UCP2 standards to quantify UCP2 in cells and isolated mitochondria after incubation of cells in different media.

**Fig. 1:**
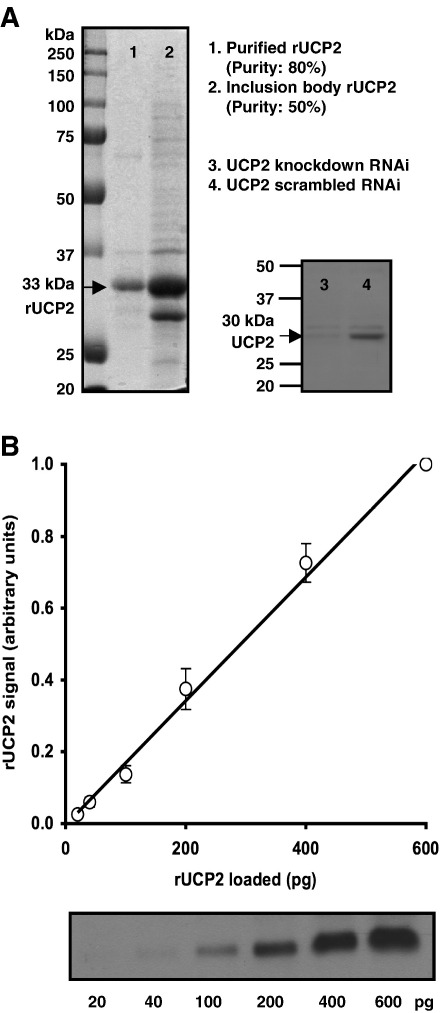
UCP2 standardisation. (A) Lanes 1 and 2: purified or unpurified inclusion body rUCP2. Purity was calculated from the intensity of the UCP2 band divided by the intensity of the whole lane. rUCP2 runs at a higher molecular weight than native UCP2 (lane 4) owing to its N-terminal appendage. Lanes 3 and 4: UCP2 antibody specificity as verified by immunoblotting INS-1E cells following treatment with siRNA against UCP2 or a scrambled siRNA control. (B) Linearity of calibration standards. rUCP2 standards were run on every gel (the immunoblot shows an example). After normalisation to the densitometry signal for 600 pg rUCP2, the densitometry signal was proportional to rUCP2 loaded. Values are means ± SEM, *n* = 12.

Since INS-1E cells are derived from rat, we generated a rat recombinant UCP2 standard. Use of a rat standard was important for accurate quantification, as the antibody (anti-human UCP2) reacted about twice as strongly with recombinant human standards than rat standards (not shown). By Gelcode^®^ Blue stain, rUCP2 in washed inclusion bodies was about 50% pure (Fig. 1A lane 2); this was increased to about 80% by using a HisTrap column (Fig. 1A lane 1); the purity was taken into account in the calibrations. The specificity of the UCP2 antibody was verified by RNAi knockdown of UCP2 in INS-1E cells (Fig. 1A lanes 3 and 4). rUCP2 was used on each immunoblot to generate an internal calibration curve. Fig. 1B shows that the densitometry signal was proportional to the rUCP2 loaded; all measurements were made within the linear range shown.

**Fig. 2:**
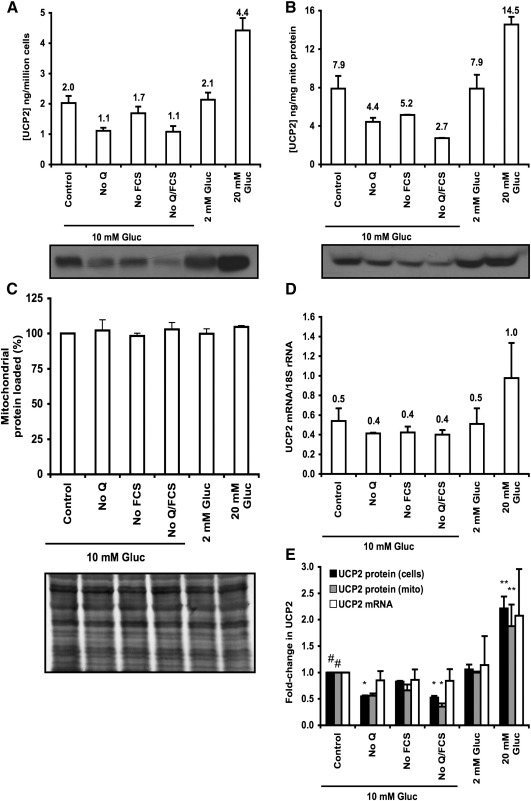
UCP2 content in INS-1E cells and mitochondria. INS-1E cells were incubated for 24 h in standard RPMI (containing 2 mM glutamine, 5% fetal calf serum and 10 mM glucose), RPMI lacking glutamine or fetal calf serum or both, or RPMI containing 2 mM or 20 mM glucose. Cell aliquots were assayed for cell and mitochondrial UCP2 content and cell *Ucp2* mRNA. (A) UCP2 protein quantified in whole cell lysates. Gel loading: 1 × 10^5^ cells/lane. Values are means ± SEM of averages of duplicate values from *n* = 3 cell preparations (the immunoblot shows an example). (B) UCP2 protein quantified in isolated mitochondria. Gel loading: 40 μg mitochondrial protein/lane. Values are means ± range of averages of triplicate values from *n* = 2 cell preparations (the immunoblot shows an example). (C) Subsequent Coomassie staining of membranes from (B) with Gelcode^®^ Blue confirmed equal protein loading. (D) *Ucp2* mRNA measured using qPCR from total mRNA and standardised against 18S rRNA. Values are means ± SEM of averages of duplicate values from *n* = 3 cell preparations. (E) Comparison of relative UCP2 protein and mRNA levels under different conditions. Values from A–C normalised to control. ⁎*P *< 0.05; ⁎⁎*P *< 0.01 compared to #. Q, glutamine; FCS, fetal calf serum; Gluc, glucose.

The UCP2 content of cells grown in standard RPMI medium, measured by calibrated immunoblot, was 2.0 ng/million cells (Fig. 2A, control bar). The UCP2 content of mitochondria isolated from the same cells was 7.9 ng/mg of mitochondrial protein (Fig. 2B, control bar). By division, the mitochondrial content of the cells was therefore 250 μg mitochondrial protein/million cells. Assuming a mitochondrial volume of 1 μl/mg protein and a cell volume of 2.4 μl/million cells (estimated from the mean diameter of trypsinised cells), mitochondria therefore comprise 11% of the volume of an INS-1E cell.

Omission of glutamine from the medium resulted in decreases in UCP2 in cells and mitochondria (Fig. 2A and B) without affecting *Ucp2* mRNA levels (Fig. 2C). The relative changes in UCP2 protein and *Ucp2* mRNA are shown in Fig. 2D. Protein content changed significantly but mRNA did not, suggesting that UCP2 is translationally controlled by glutamine in INS-1E cells, in agreement with studies in other cells [19,20]. Serum starvation had no effect on *Ucp2* mRNA, and although it consistently lowered UCP2 protein levels, implying translational control, this effect was not significant (Fig. 2D).

Lowering glucose to 2 mM had no effect on UCP2 protein or mRNA, but doubling glucose concentration to 20 mM increased protein levels 2-fold over control (Fig. 2D), and affected mRNA in the same direction, although not significantly, suggesting transcriptional control of *Ucp2* mRNA by high glucose in INS-1E cells. This is explained by [28], where increased glucose was shown to transcriptionally activate *Ucp2* with a lack of reversibility of *Ucp2* mRNA levels when glucose in the medium is changed from a higher to a lower concentration.

**Fig. 3:**
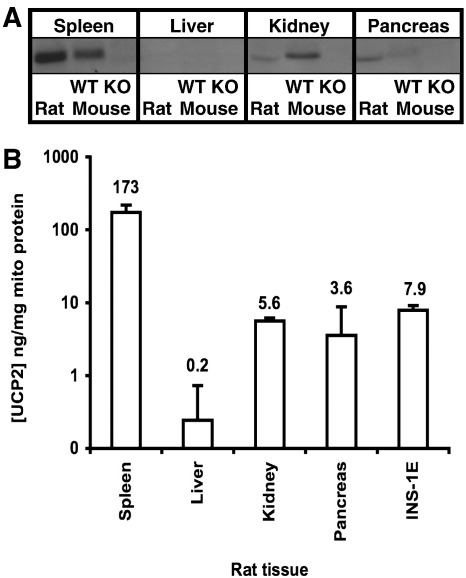
UCP2 content in rodent tissue mitochondria. (A) UCP2 immunoblots of rat and mouse mitochondria from different tissues. Mitochondrial protein loaded: 5 μg spleen, 40 μg liver, 30 μg kidney, 40 μg pancreas. (B) Quantification of UCP2 in mitochondria from rat tissues using rUCP2 standards. Values are means ± range of averages of duplicate values from *n* = 2 mitochondrial preparations.

Between them, these interventions caused UCP2 concentrations to vary 4–5-fold in 24 h, from 1.0–4.4 ng/million cells (2.7–14.5 ng/mg mitochondrial protein). These mitochondrial concentrations are hundreds of times lower than UCP1 in brown adipose tissue, and 20-fold lower than UCP3 in skeletal muscle [29]. To compare them to UCP2 concentrations in other rat tissues, we analysed mitochondria from rat spleen, liver, kidney and pancreas. Fig. 3A shows that the antibody detected UCP2 in spleen, kidney and pancreas mitochondria from wild-type mice and rats, but not in *Ucp2*^−/−^ mice. Fig. 3B shows the values for rat mitochondria, quantified as before. Levels of UCP2 in INS-1E cells were similar to those in kidney and whole pancreas, and 20-fold lower than in spleen, previously reported to have the highest UCP2 levels of any tissue [29].

### 3.2 UCP2 has a short half-life in INS-1E cells

**Fig. 4:**
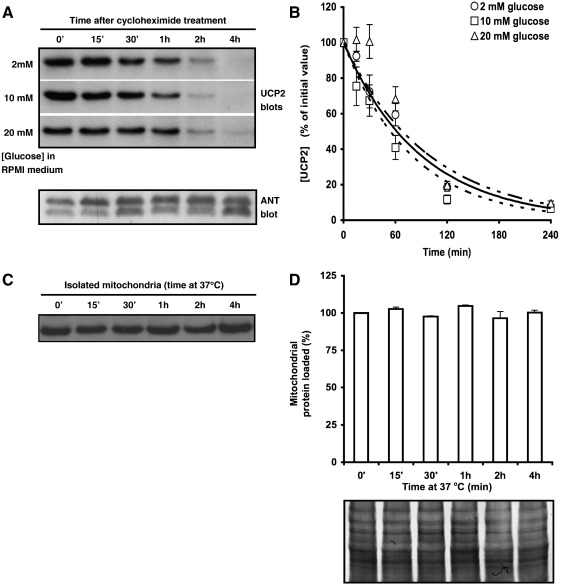
UCP2 half-life. (A) INS-1E cells were incubated for 24 h in RPMI containing 2 mM, 10 mM, or 20 mM glucose, then treated with 10 μg/ml cycloheximide to arrest protein synthesis, harvested at the times shown and immunoblotted for UCP2. Stripped membranes were reblotted for ANT as a control (the lower immunoblot shows an example). Gel loading: 1.5 × 10^5^ cells/lane. (B) Cell UCP2 content (normalised to time zero in each condition) as a function of time after cycloheximide treatment. Values are means ± SEM from *n* = 4 mitochondrial preparations. (C) Mitochondria were isolated from cells incubated for 24 h in standard RPMI, incubated in SHE buffer at 37 °C for various times then immunoblotted for UCP2. Gel loading: 36 μg mitochondrial protein/lane. (D) Subsequent Coomassie staining of membranes from (C) with Gelcode^®^ Blue confirmed equal protein loading.

To investigate the role of protein degradation on the variation of UCP2 levels, INS-1E cells were treated with cycloheximide to arrest protein synthesis, and UCP2 levels were measured at different times by immunoblotting. UCP2 was found to have a short half-life of approximately 1 h (Fig. 4A, B), presumably allowing the dynamic fluctuations in UCP2 seen above and perhaps accounting for the low abundance of this protein. This short half-life contrasts with the much longer half-life of other members of the mitochondrial carrier family, such as UCP1 (see [23,30]) and ANT (Fig. 4A). Furthermore, the half-life of UCP2 does not appear to be dependent on protein levels because doubling UCP2 content by incubation of INS-1E cells with 20 mM glucose had no effect on protein turnover, and expression of UCP1 and UCP2 in the same yeast system still gives a much longer half-life for UCP1 (30 h) than UCP2 (30 min) [23].

In contrast to whole cells, UCP2 levels were stable in isolated mitochondria (Fig. 4C), implying that either the signal for degradation or degradation itself occurs outside the mitochondrion.

Our work shows that owing to the short half-life (1 h) of UCP2, incubating cells in simple salt media can result in low UCP2 levels. This work may explain the variability of UCP2-mediated effects in different laboratories. Reassuringly, UCP2 is stable once mitochondria have been isolated.

## 4 Conclusion

There is dynamic regulation of UCP2 concentration in INS-1E cells that is characterised by fast rates of synthesis and degradation. UCP2 synthesis is under translational and probably transcriptional control (as literature suggests for other cells [19,20,28]), and its dynamic regulation is enabled by very rapid protein turnover. This highlights the dynamic response of UCP2 concentration and perhaps activity to changes in the nutrient supply, and sheds light on the ability of UCP2 to attenuate insulin secretion by uncoupling in INS-1E cells [6], beta-cells from pancreatic islets [31], and whole animals [4,5].
